# Neuropsychiatric Systemic Lupus Erythematosus Presenting With Striatal Lupus Encephalitis and Catatonia: A Multidisciplinary Diagnostic Challenge

**DOI:** 10.7759/cureus.112480

**Published:** 2026-07-11

**Authors:** Edward Olufunmilayo, Asaad Hamed, Raheel Mushtaq, Amelia Gledhill, Olabambo Ogunbambi

**Affiliations:** 1 Rheumatology, Hull University Teaching Hospitals NHS Trust, Hull, GBR; 2 Psychiatry, Hull University Teaching Hospitals NHS Trust, Hull, GBR

**Keywords:** autoimmune neuroinflammation, basal ganglia disease, catatonia, neuroinflammation and mental health, neuropsychiatric sle, sle

## Abstract

Neuropsychiatric lupus represents one of the most complex and heterogeneous manifestations of systemic lupus erythematosus (SLE). Presentations may include cognitive dysfunction, mood disorders, psychosis, seizures, and movement disorders, often mimicking primary neurological or psychiatric conditions. We report the case of a woman with established SLE who developed acute behavioural change, cognitive decline, and psychomotor agitation progressing to catatonia. Neuroimaging demonstrated symmetrical basal ganglia abnormalities with associated cortical and basal ganglia microinfarcts. Extensive investigation excluded infectious, embolic, and primary neurodegenerative causes. The clinical picture was ultimately attributed to striatal lupus encephalitis, a rare manifestation of neuropsychiatric SLE (NPSLE). She was treated with high-dose corticosteroids and intravenous cyclophosphamide with subsequent clinical improvement, while catatonic symptoms responded rapidly to benzodiazepine therapy. Repeat MRI showed partial regression of basal ganglia abnormalities but new microinfarcts. This case highlights the evolving nature of NPSLE, the importance of multidisciplinary collaboration, and the need to consider autoimmune mechanisms in atypical neuropsychiatric presentations.

## Introduction

Systemic lupus erythematosus (SLE) is a chronic multisystem autoimmune disorder characterised by the production of autoantibodies and immune-mediated tissue damage. Neuropsychiatric involvement occurs in approximately 30-40% of patients and encompasses a wide spectrum of neurological and psychiatric manifestations collectively termed neuropsychiatric systemic lupus erythematosus (NPSLE) [1]. Given the multifaceted symptoms, the unpredictability of onset of presentation relative to the SLE disease course, and the gaps in the understanding of its pathogenesis, NPSLE can be quite challenging to diagnose. 

The American College of Rheumatology recognises 19 different neuropsychiatric syndromes associated with SLE [2]. The pathogenesis is multifactorial and may involve inflammatory mechanisms, autoantibody-mediated neuronal injury, microvascular thrombosis, or cytokine-driven neurotoxicity [3]. Among these manifestations, basal ganglia involvement and catatonia are rare but increasingly recognised. As highlighted in this report, there are a handful of case reports that have described evidence of striatal involvement in NPSLE, but more epidemiological studies are needed to truly quantify its rarity.

Catatonia is a complex neuropsychiatric syndrome of both hypo- and hyperkinetic movement and behavioural abnormalities, often associated with many psychiatric and organic brain conditions. Psychomotor signs range from stupor to agitation and include pathognomonic features such as verbigeration and waxy flexibility. Severe and complicated cases often result in autonomic instability (malignant catatonia), which can be fatal if unrecognised and untreated [4].

The basal ganglia and related nuclei consist of a number of cell groups within the cerebrum and brainstem engaged primarily in motor control, and other roles such as motor learning, executive functions and behaviour. The core components of the basal ganglia include the striatum and globus pallidus, which have close functional associations with the subthalamic nucleus, substantia nigra and ventral thalamic nuclei. The striatum consists of the caudate nucleus and putamen. Functional imaging studies in catatonic patients have shown dysfunction in the supplementary motor area (SMA), thalamus and the basal ganglia [5].

Striatal lupus encephalitis represents an uncommon form of NPSLE characterised by bilateral basal ganglia abnormalities and behavioural or movement disturbances. Previously reported cases demonstrate the rarity of striatal involvement and emphasise the importance of early immunosuppressive therapy. Catatonia, although well described in psychiatric disorders, is rarely reported in SLE and may be overlooked in complex patients [6].

We describe a rare case with a peculiar combination of striatal lupus encephalitis with catatonia, a prominent psychiatric prodrome, cortical microinfarcts, and documented temporal evolution of neuroimaging findings.

## Case presentation

A West African woman in her 30s, with established SLE, presented to our tertiary facility for a planned belimumab infusion. Her SLE had previously involved cutaneous, musculoskeletal, haematological, and constitutional manifestations, and she was receiving low-dose corticosteroid therapy. She had presented three years earlier with fever and severe generalised erythematous rash on sun-exposed areas, with rapid spread from the arms to the face, neck, legs and trunk. The rash evolved into vesicles and large, tense haemorrhagic bullae (Figure 1). There were no mucosal lesions. Skin biopsy demonstrated histological features consistent with toxic epidermal necrolysis (TEN), and she was diagnosed as a case of TEN-like acute cutaneous lupus [7]. She was managed with IV methylprednisolone and rituximab. She developed severe denudation of the skin with an epidermal loss of more than 85% total body surface area (TBSA), and this was thought to have possibly been contributed to by an adverse reaction to rituximab or hydroxychloroquine. She was managed in the regional burns unit and made a full recovery.

**Figure 1:**
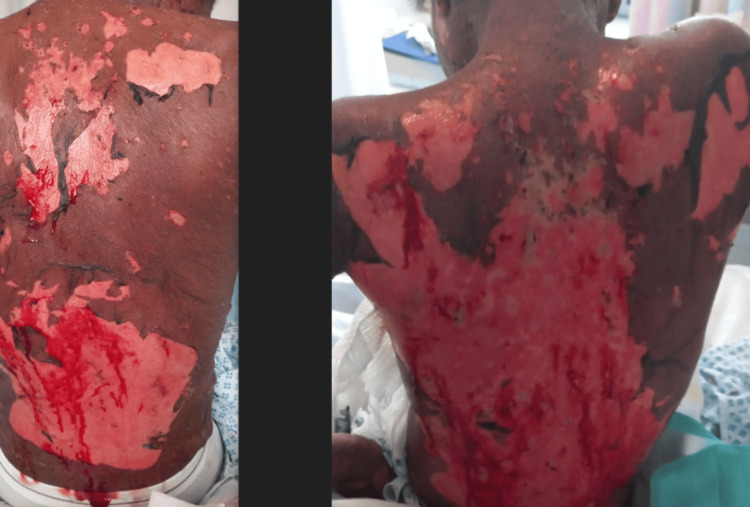
Clinical images from the patient's presentation with TEN-like acute cutaneous lupus three years earlier, showing ruptured large haemorrhagic blisters and bullae TEN: toxic epidermal necrolysis

From about four weeks prior to this new admission, she had experienced malaise, weight loss, reduced appetite, and mild cough. Within the first week of admission, her husband reported new behavioural changes, including disorganised communication, inappropriate telephone calls to neighbours, and incoherent text messages. There were also reports of escalating psychomotor restlessness characterised by constant pacing and an inability to settle. This progressed to refusal of food, fluids, and medication. Episodes of behavioural disinhibition were observed, including inappropriate removal of clothing, without aggression. Clinical examination revealed lethargy, poor affect, and cognitive slowing but no focal neurological deficit.

On assessment by the Mental Health Liaison Service, she described intense subjective inner restlessness and anxiety. Mood was reported as “fine”, and she denied depressive symptoms, suicidal ideation, or psychotic experiences. She was fully oriented with no evidence of hallucinations, delusions or formal thought disorder. Engagement was limited by marked psychomotor agitation. Subsequently, clear catatonic features soon emerged, including echolalia, verbigeration, palilalia, stereotypy and mannerisms. Mannerisms were characterised by unusual hand movements over the abdomen. She had suffered from shingles affecting the abdominal wall months prior to this presentation, and so it was unclear whether the mannerisms were purely related to pain from possible post-herpetic neuralgia, or associated with the other catatonic features displayed. No new vesicular rash was noted on this admission. A Bush-Francis catatonia score [8] of 20 was recorded. As no associated hallucinations, delusions or thought disorders were noted, it was concluded that the catatonic features were unlikely to be due to a primary psychotic illness.

Investigations

Magnetic resonance imaging (MRI) of the brain demonstrated striking symmetrical hyperintensity and swelling of the corpora striata on T2-weighted imaging with relative sparing of the posterior putamina(Figure 2). Additional abnormalities included multiple small cortical diffusion-restricted lesions in both cerebral hemispheres, suggestive of acute microinfarcts. Cardiac evaluation, including transthoracic echocardiography and cardiac MRI(Figure 3), revealed no embolic source. No arterial occlusion or vasculitic changes were observed on MR angiography.

**Figure 2:**
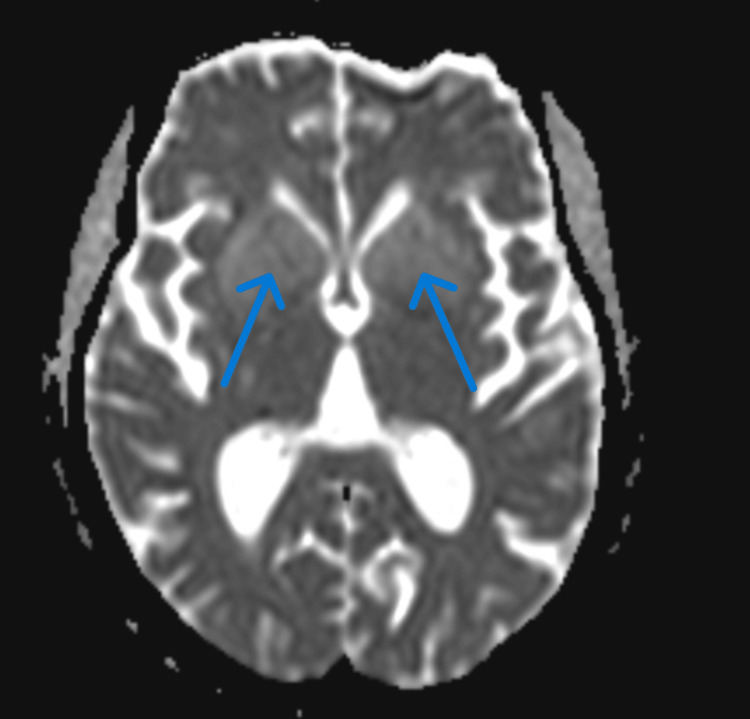
T2-weighted MRI of the brain showing symmetrical hyperintensity and swelling of the corpora striata, with relative sparing of the posterior putamina and effacement of the anterior horns of the lateral ventricles Imaging technique: sagittal T2W FLAIR 3D TSE, axial T2W TSE (BLADE), DWI and 3D TOF MRA T2W: T2-weighted; FLAIR: fluid-attenuated inversion recovery; TSE: turbo spin echo; DWI: diffusion-weighted imaging; TOF: time of flight; MRA: magnetic resonance angiography

**Figure 3:**
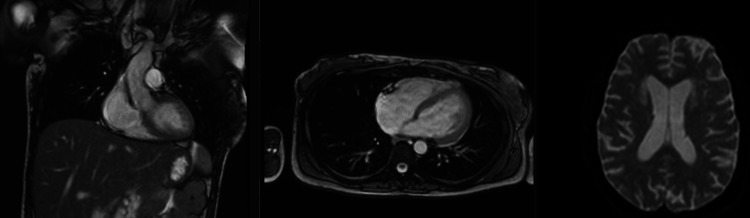
Coronal and axial views of essentially normal cardiac MRI without intracardiac thrombi, and MR brain angiography without occlusive or vasculitic features

C-reactive protein was elevated at 32 mg/L. Full blood counts showed anaemia (haemoglobin 83 g/L) and mild lymphopaenia (0.61 X 10^9^/L). Infection screens, including urine MCS (microscopy, culture and sensitivity), sputum MCS, respiratory PCR panel and chest radiographs were unremarkable. Complement levels were normal. Autoimmune panel showed elevated Sm IgG Abs (109.8U); Ro-60 Abs >583.27U; RNP-68 Abs >181.99U (baseline positivity) and normal levels of Anti dsDNA (18.8 U/mL). Importantly, we noted that anti-dsDNA levels had been significantly elevated exactly two months prior to this index presentation (147.2 U/mL), associated with reduced C3 complement (0.75 g/L), although she had no specific symptoms to suggest a lupus flare at that time. Anti-phospholipid antibody tests were negative. Urine protein-creatinine ratio showed no significant proteinuria. Renal and liver function tests were normal. Cerebrospinal fluid analysis showed no evidence of infection, and the N-methyl-D-aspartate (NMDA) receptor antibody assay was negative(Table 1). Given the patient's mild cough, an associated viral infection may explain the elevated CRP, although the routine respiratory PCR panel was negative.

**Table 1:** Relevant laboratory investigations at presentation RNP: ribonucleoprotein; dsDNA: double-stranded deoxyribonucleic acid; NMDA: N-methyl D-aspartate; ALT: alanine aminotransferase

Investigation	Value at presentation	Reference range
C-reactive Protein	32 mg/L	0-5 mg/L
Haemoglobin	83 g/L	120-160 g/L
White cell count	5.3 X 10^9 ^/L	4.0-11.0 X 10^9 ^/L
Lymphocyte count	0.61 X 10^9 ^/L	0.8-3.4 X 10^9 ^/L
C3 complement	1.33 g/L (0.75 g/L two months earlier)	0.9-1.8 g/L
C4 complement	0.33 g/L	0.1-0.4 g/L
Smith IgG Antibodies	109.8 U	0.0-4.9 U
Anti Ro-60	>583.27 U	0.0-29.9 U
Anti RNP-68	>181.99 U	0.0-29.9 U
Anti-dsDNA	18.8 U/mL (147.2 U/mL Two months earlier)	0.0-26.9 iU/mL
NMDA receptor antibodies	Negative	Negative
Creatinine	67 umol/L	55-87 umol/L
ALT	28 IU/L	5-45 IU/L
Urine protein: creatinine ratio	17 mg/mmol	<15mg/mmol
Anti-B2 glycoprotein 1 antibodies	11 CU	0.0-19.9 CU
Anti-cardiolipin antibodies	6.3 CU	0.0-19.9 CU

The SLEDAI (Systemic Lupus Erythematosus Disease Activity Index) score came to 9, indicative of a mild or moderate flare of lupus. This patient had normal levels of the usual serological markers of lupus flare (e.g., complement, anti-dsDNA) at presentation. It is known that these markers may remain normal at the time a flare becomes clinically apparent, but a preceding elevation in anti-dsDNA levels by weeks to months may predict an oncoming disease flare [9]. This may have been the case in our patient.

Differential diagnoses

The radiological differential diagnosis included metabolic or toxic encephalopathy, Creutzfeldt-Jakob disease, and autoimmune encephalitides like anti-NMDA receptor encephalitis. However, the clinical context of active SLE with the outlined clinical features raised suspicion for NPSLE. Given the absence of alternative explanations, the neurology, rheumatology and psychiatry teams concluded that the presentation was most consistent with striatal lupus encephalitis with catatonia.

Treatment

International guidelines [10] for the treatment of severe (including neuropsychiatric) manifestations of SLE include induction with high-dose (oral or IV) steroid alone or in combination with either high-dose azathioprine, cyclophosphamide, mycophenolate mofetil or ciclosporin. Maintenance therapy is typically with lower doses of prednisolone with either mycophenolate, azathioprine or ciclosporin plus hydroxychloroquine. There is, however, a limited evidence base for specific treatments or guidelines for neuropsychiatric SLE. Rituximab and hydroxychloroquine were not considered for this patient due to a history of suspected adverse reaction, and she was ineligible for azathioprine due to low serum levels of thiopurine methyltransferase (TPMT). Cyclophosphamide and mycophenolate had been previously avoided in her previous presentations, as she was trying to conceive. 

Given the severity of this presentation, and considering the highlighted issues regarding treatment options, she received IV methylprednisolone 500 mg daily for five days, followed by high-dose oral prednisolone (started at 1 mg/kg and tapered gradually over months to a maintenance dose of 5 mg daily). Intravenous cyclophosphamide (15 mg/kg) was initiated, and she completed 6 cycles in total, at 2-week intervals. As advised by the fertility clinic, she had monthly injections of Leuprorelin to minimise the effect of these treatments on fertility. On account of the brain microinfarcts, she was also started on antiplatelet therapy (clopidogrel 75 mg daily) as advised by the stroke team.

Following two cycles of cyclophosphamide, a modest improvement in behavioural symptoms was observed. Lorazepam was titrated with additional PRN dosing following discussion of risks and benefits, and resulted in the resolution of catatonia. Quetiapine, which had initially been commenced on the ward, was discontinued by the psychiatry team due to the absence of psychotic features and the potential to worsen catatonia.

Outcome and follow-up

Over the course of about five weeks, anxiety and pacing gradually improved. Lorazepam was carefully titrated, and catatonic symptoms resolved completely. She was then transferred to the rehabilitation ward. Once clinically stable, lorazepam was gradually tapered, and at discharge, she remained on 1 mg twice daily.

During admission, she also reported significant abdominal pain at the site of previous shingles, possibly related to post-herpetic neuralgia. This was managed with topical capsaicin, lidocaine patches and duloxetine. She subsequently described unusual internal sensations affecting her abdomen and legs, initially raising concern for further neuropsychiatric involvement. These symptoms were attributed to duloxetine, and dose reduction resulted in complete resolution.

Repeat MRI performed after three months of treatments demonstrated interval regression of the previously noted basal ganglia swelling and T2 signal abnormalities (Figure 4). However, new areas of diffusion restriction were present within the basal ganglia and corona radiata, consistent with additional microinfarctions. A multidisciplinary neurology meeting concluded that these lesions were most consistent with SLE-related pathology rather than embolic stroke. Although the significant interval regression in the basal ganglia abnormalities was a good radiological marker of improvement, it was unclear why additional microinfarcts were present. As there were two broad pathologies on imaging (inflammatory and vascular), this may reflect a lag in the response of the vascular pathology to the immunosuppression.

**Figure 4:**
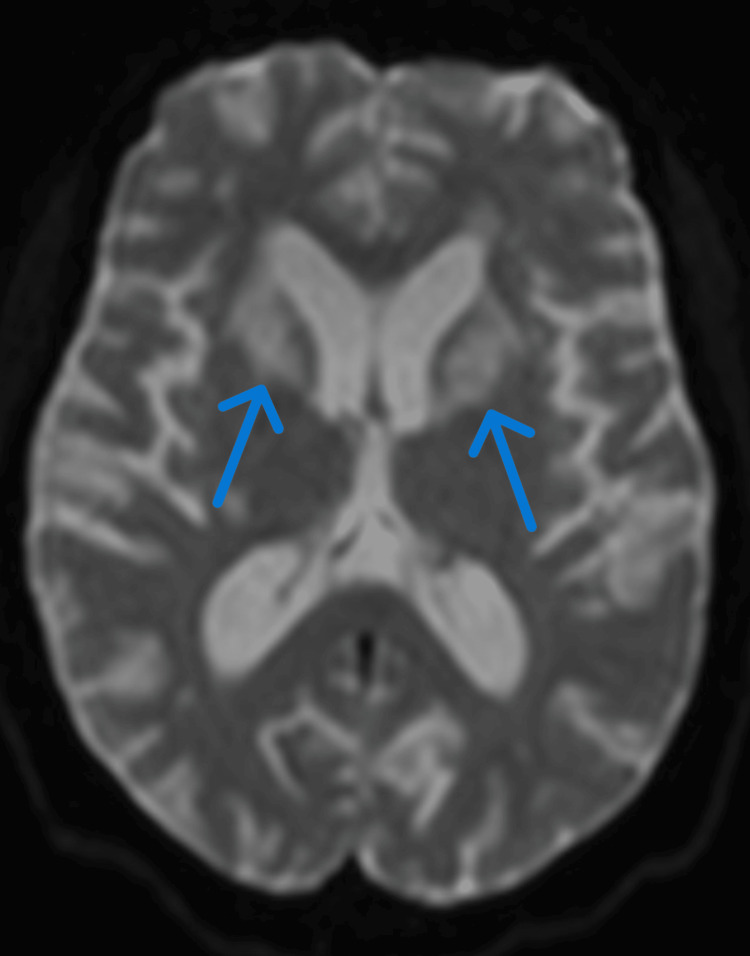
Three-month interval MRI brain showing significant regression of the previously noted basal ganglia swellings and T2 signal abnormalities; effacement of the anterior horns of the lateral ventricles is also resolved

About four months into admission, neuropsychological assessment using the Repeatable Battery for the Assessment of Neuropsychological Status (RBANS) during admission demonstrated impairments across multiple domains, including memory, attention, language, and executive functioning, consistent with lupus-related cerebral involvement. She had a very limited understanding of her presentation and functioning and lacked the capacity to make certain treatment decisions.

At the point of discharge, five months post-admission, home leave was trialled with appropriate outcomes; she was able to manage her own medications, with some support required in the home environment. Family also reported some degree of apathy and anxiety at home in the weeks following discharge. Mild ongoing risks of self-neglect and vulnerability were noted, related to cognitive impairment rather than active psychiatric symptoms, and appropriate safety planning was implemented.

She was discharged home with a gradual taper of lorazepam under general practitioner supervision, continued duloxetine pending review, and follow-up arranged with the Community Mental Health Team, Rehabilitation and Rheumatology services. Maintenance immunosuppression using mycophenolate mofetil 500 mg thrice daily was started.

## Discussion

Neuropsychiatric involvement in SLE is highly heterogeneous and remains a diagnostic challenge. Manifestations may arise from inflammatory, thrombotic, or antibody-mediated processes affecting the central nervous system [11-13]. The clinical spectrum includes cognitive impairment, psychosis, seizures, cerebrovascular disease and movement disorders, and these symptoms frequently mimic primary neurological or psychiatric conditions [11,13].

In the index case, the patient developed behavioural disturbance, cognitive decline and psychomotor agitation before progressing to catatonia, associated with bilateral basal ganglia abnormalities on MRI. This constellation of findings is consistent with striatal lupus encephalitis with catatonia, a rare but recognised manifestation of NPSLE.

Basal ganglia involvement in NPSLE

Basal ganglia abnormalities are uncommon in lupus but have been described in association with movement disorders, psychiatric symptoms and encephalopathy, and proposed mechanisms include immune-mediated neuronal injury, small-vessel vasculopathy and micro-thrombotic disease [14,15].

Some case reports have documented symmetrical basal ganglia signal changes in lupus patients presenting with neuropsychiatric symptoms. In many cases, these abnormalities resemble metabolic encephalopathies or autoimmune encephalitis, complicating the diagnosis [16,17].

The imaging findings in our patient were notable for symmetrical striatal involvement accompanied by multiple cortical and basal ganglia microinfarcts. The absence of vasculitic features on angiography and the lack of an embolic source suggested microvascular disease related to lupus activity.

Catatonia in NPSLE

Early features in this patient, including pacing, inability to settle, and intense inner restlessness, could easily have been misattributed to primary anxiety, medication side effects, or delirium. The subsequent emergence of verbigeration and stereotyped movements clarified the diagnosis of catatonia. This evolving presentation underscores the need for ongoing re-evaluation in medically complex patients, particularly when symptoms do not follow expected patterns.

Catatonia is rarely reported in SLE but represents an important clinical entity because it responds rapidly to benzodiazepine therapy [15-18]. Several mechanisms have been proposed, including inflammatory disruption of GABAergic and glutamatergic pathways and immune-mediated neuronal dysfunction [4,19,20].

A review of published cases suggests that catatonia may occur during active lupus disease or as part of neuropsychiatric lupus. In most cases, symptoms improve with benzodiazepines and immunosuppressive therapy. In this patient, catatonia developed several weeks after the initial neuropsychiatric symptoms and responded dramatically to lorazepam. Recognition of this syndrome was crucial to avoiding potential misdiagnosis as a primary psychiatric illness.

Comparison with previously reported cases

Striatal lupus encephalitis has been described only rarely in the literature, and the associated development of catatonia reflects the uniqueness of this specific case. Most reported patients present with behavioural disturbances, movement disorders or cognitive impairment, often accompanied by bilateral basal ganglia abnormalities on neuroimaging. Treatment typically involves corticosteroids and immunosuppressive therapy with favourable outcomes in many cases.

Compared with previously reported cases, our patient demonstrated several distinctive features, namely, prominent behavioural and cognitive symptoms preceding neurological findings, development of catatonia during the disease course and the presence of cortical and basal ganglia microinfarctions, suggesting overlapping inflammatory and vascular mechanisms (Table 2). These findings illustrate the complex, multifactorial and often evolving nature of neuropsychiatric lupus.

**Table 2:** Comparison of reported cases of catatonia in systemic lupus erythematosus and striatal lupus encephalitis IVMP: intravenous methylprednisolone

Case report [Reference]	Age/Sex	Clinical presentation	Neuroimaging findings	Treatment	Outcome
[16]	20/F	Catatonia, psychosis	Normal MRI	IVMP, Cyclophosphamide, Lorazepam	Improvement in Catatonia
[17]	28/F	Aseptic meningitis	Bilateral basal ganglia hyperintensities	Immunosuppresion	Clinical improvement
[21]	68/F	Cognitive impairment, weakness	Basal ganglia hyperintensities	IVMP, Plasmapheresis	Clinical improvement
[22]	32/F	Neuropsychiatric lupus with catatonia	MRI not done	IVMP, Cyclophosphamide	Resolution of catatonia
Present case	35/F	Behavioural change, cognitive decline, catatonia	Symmetrical basal ganglia abnormalities with cortical and basal ganglia microinfarcts	IVMP, Cyclophosphamide, Lorazepam	Clinical improvement, resolution of catatonia

Other psychiatric considerations

Cognitive impairment was prominent, affecting memory, attention, language, and executive functioning. Such deficits in SLE are frequently under-recognised and may significantly impact functional independence and employment [23-25]. Neuroimaging and histopathological studies support cerebral involvement in NPSLE patients who develop cognitive impairments, including microinfarction and inflammatory changes [26], as seen in this case.

Review about seven weeks post-discharge demonstrated some degree of apathy and low mood as noted by the patient’s partner. While these may be related to the NPSLE, the psychosocial and cultural context of losing her job soon after discharge, and ongoing struggles with conception are important considerations and potential explanations for the low mood. It is also known that mood disorders, on their own, and indeed, within the context of NPSLE, often demonstrate long recovery times [27].

Therapeutic considerations

The management of NPSLE depends on the presumed underlying mechanism. Inflammatory manifestations are typically treated with corticosteroids and immunosuppressive agents such as cyclophosphamide, azathioprine, ciclosporin, mycophenolate mofetil, IV immunoglobulins, plasmapheresis or biologics [10]. In this case, aggressive immunosuppression with corticosteroids and cyclophosphamide was associated with clinical improvement and regression of basal ganglia abnormalities on follow-up imaging. Further treatments, such as IV immunoglobulins, plasmapheresis or biologics, could have been considered if symptoms and signs were refractory to the initial treatments. Catatonia responded rapidly to benzodiazepines, consistent with previous reports [8,18]. Early recognition of catatonia is crucial because untreated cases may progress to severe complications, including autonomic instability or malignant catatonia.

This case demonstrates the evolving and multi-layered nature of NPSLE and the importance of early recognition, careful longitudinal assessment, and individualised multidisciplinary management in optimising outcomes and supporting functional recovery.

Limitations

Although, as previously noted, catatonia has been reported to be associated with basal ganglia dysfunction, it is still not possible in this case, as with many cases, to fully attribute the patient's catatonic features to the imaging abnormalities seen in the basal ganglia with definite certainty. This is due to the significant variety and complexities of the causes of catatonia itself, the complex manifestations of basal ganglia pathologies, the varying manifestations of NPSLE, and the dearth of epidemiological data and knowledge of the pathophysiology of striatal lupus encephalitis. In terms of ruling out other potential mimics, an electroencephalogram may have been useful in excluding a non-convulsive status epilepticus, although this was deemed clinically unlikely.

## Conclusions

Neuropsychiatric systemic lupus erythematosus (NPSLE) remains a complex and unique diagnostic and, indeed, therapeutic challenge. This case highlights an unusual presentation involving striatal lupus encephalitis and catatonia with characteristic basal ganglia abnormalities on MRI. This case also underscores several important clinical lessons. NPSLE can present with prominent psychiatric symptoms before neurological features emerge. Catatonia with basal ganglia involvement is a rare but treatable manifestation of NPSLE, as demonstrated in this particular case. However, the relative quantitative contributions of inflammation and microvascular disease to the pathophysiologic processes in NPSLE remain uncertain.

The recognition of atypical presentations is essential to ensure timely diagnosis and treatment, and this case suggests that early multidisciplinary evaluation and aggressive immunosuppressive therapy can improve patient outcomes to an appreciable degree.
